# Is sentinel lymph node frozen section analysis necessary in hormone receptor–positive, HER2-negative, clinically node-negative breast cancer patients undergoing upfront mastectomy?

**DOI:** 10.3389/fonc.2026.1792989

**Published:** 2026-03-18

**Authors:** Bülent Aksel, Mehmet Furkan Sağdıç, Lutfi Doğan

**Affiliations:** Department of Surgical Oncology, Ankara Etlik City Hospital, Ankara, Türkiye

**Keywords:** breast cancer, mastectomy, multidisciplinary management, permanent section, sentinel lymph node biopsy

## Abstract

**Background:**

Axillary surgical management in breast cancer has recently shifted toward less invasive approaches. Although sentinel lymph node biopsy (SLNB) remains the standard method for axillary staging in clinically node-negative patients, the necessity of performing intraoperative frozen section (IFS) in those undergoing up-front mastectomy continues to be a matter of debate.

**Method:**

In this retrospective study, we evaluated 47 women aged 18 years or older who were diagnosed with hormone receptor–positive, HER2-negative cT1–3 breast cancer patients and underwent upfront mastectomy combined with SLNB between October 2022 – January 2026. Our analyses excluded patients who received neoadjuvant therapy, underwent breast-conserving surgery, or demonstrated clinical or radiological evidence of nodal involvement. SLNB was performed using a dual-agent technique. In line with institutional routine practice, SLNB were assessed on paraffin-embedded permanent sections rather than IFS analysis. We analyzed treatment processes together with clinical and pathological variables.

**Results:**

Among 47 patients, 39 (83%) were node-negative, while 8 (17%) were node-positive. There were no significant differences between the groups by age, tumor size, histopathologic subtype. Lymphovascular invasion and extracapsular extension were significantly more prevalent in SLNB-positive patients (p = 0.021). None of the node-positive patients underwent axillary dissection; instead, a multidisciplinary tumor board decision was to maintain treatment with axillary radiotherapy. The absence of IFS analysis neither altered therapeutic planning nor necessitated any additional surgical intervention. There were no regional recurrences after a median follow-up of 27 months.

**Conclusion:**

In this retrospective cohort of clinically node-negative, hormone receptor–positive, HER2-negative cT1–3 breast cancer patients undergoing upfront mastectomy, omission of intraoperative frozen section (IFS) analysis was not associated with early regional recurrence during the observed follow-up. Routine IFS would likely have altered immediate surgical management in only a small minority of patients in this selected population, suggesting limited clinical utility in similar low-risk cohorts.

## 1 Introduction

Surgical management of the axilla used to be regarded as a key critical determinant of prognosis in breast cancer. However, evidence has shown that axillary lymph node dissection (ALND) could serve primarily as a staging and prognostic tool rather than a means of preventing distant metastasis. The shift toward less extensive axillary surgery was initiated by the NSABP B-04 trial (1). Later, the NSABP B-32 trial demonstrated that sentinel lymph node biopsy (SLNB) could be performed safely (2). Subsequently, the ACOSOG Z0011 trial revealed that axillary lymph node dissection (ALND) did not offer superior regional control or survival compared with SLNB alone among patients with cT1–2 disease undergoing breast-conserving surgery (BCS) who had one or two positive sentinel lymph nodes (SLN) (3, 4). The publication of the SENOMAC trial results in 2024 further confirmed that ALND can be safely omitted with nodal irradiation in T1–T3 cN0 patients undergoing mastectomy with one or two positive SLNs (5).

Similarly, the Dutch BOOG 2013–07 trial reported no difference in 5-year regional recurrence–free survival between mastectomy patients with SLN metastasis who received no additional axillary treatment and those who underwent ALND or axillary radiotherapy (6). Another key study, the Hungarian OTOASOR phase III non-inferiority trial, included patients in whom two or more SLNs were dissected. Individuals with malignant SLNs were randomized to either axillary dissection or regional nodal irradiation. After an eight-year follow-up, the two groups demonstrated comparable rates of axillary recurrence (2% vs. 1.7%) (7).

Despite these advances, recommendations for axillary management remain clearly defined in current clinical guidelines (8, 9). In patients with clinically node-negative (cN0) invasive breast cancer measuring ≤5 cm who undergo mastectomy, completion axillary lymph node dissection (ALND) can be avoided when one or two positive sentinel lymph nodes (SLNs) are identified, provided that postmastectomy radiotherapy (PMRT) including regional nodal irradiation (RNI) is planned. However, when nodal involvement is more extensive—particularly in cases with three or more positive lymph nodes—completion ALND followed by PMRT is generally advised. At the same time, the extent of nodal disease, tumor biology, and other clinicopathologic features should be considered in treatment planning. Although recent trials have explored further de-escalation of axillary surgery, omission of ALND in patients with ≥3 positive nodes have not yet become standard practice and continues to be debated. For this reason, management decisions should be individualized and discussed within a multidisciplinary setting.

The shifting approach to axillary management has also led to revisiting the role of IFS analysis. Beyond its inherent limitations (e.g., false-negative/positive results, increased costs, and prolonged operative time), IFS analysis may also lead to unnecessary additional axillary treatment. In our previous report, we noted that our institution transitioned from IFS analysis to permanent section analysis for BCS patients (10). Ultimately, the present study aims to assess whether IFS analysis is essential in clinically node-negative, hormone receptor–positive, HER2-negative cT1–3 patients undergoing upfront mastectomy.

## 2 Materials & methods

In this study, we retrospectively analyzed data from women aged 18 years or older with hormone receptor-positive and HER2-negative subtype of cT1–3N0 breast cancer who underwent upfront mastectomy and SLNB at the Surgical Oncology Department of Ankara Etlik City Hospital, University of Health Sciences, between October 2022 and January 2026. Our analyses excluded data from patients who had received neoadjuvant therapy, those in whom SLNB evaluation was not performed or SLNs could not be identified intraoperatively, those undergoing breast-conserving surgery, patients with clinical suspicion of nodal metastasis or confirmed cN1 disease, those with recurrent breast cancer, those operated on for ductal carcinoma *in situ*, as well as patients with HER2-positive or triple-negative breast cancer. The indication for neoadjuvant therapy was determined by multidisciplinary tumor board assessment based on tumor biology, clinical stage, and surgical operability. Patients undergoing breast-conserving surgery were excluded to ensure a homogeneous surgical cohort and to avoid potential confounding related to differences in intraoperative decision-making and adjuvant treatment strategies between mastectomy and breast-conserving surgery. The outcomes of omitting intraoperative frozen section analysis in patients treated with breast-conserving surgery at our institution have previously been reported (8). Therefore, the present study was specifically designed to evaluate this issue specifically in the upfront mastectomy setting. Axillary status was routinely assessed by preoperative ultrasonography (USG). SLNB was performed using dual-agent mapping with isosulfan blue dye and radioisotope. In all cases, intraoperative SLNB evaluation was omitted, and nodal assessment was completed using paraffin-embedded permanent sections. During the study period, intraoperative frozen section analysis was not part of our institutional routine for clinically node-negative patients undergoing upfront mastectomy; therefore, sentinel lymph nodes were assessed on permanent paraffin sections. All sentinel lymph nodes were fixed in 10% neutral-buffered formalin and processed for routine paraffin embedding. Each node was measured and grossly sliced at ≤2-mm intervals along its short axis to ensure complete tissue representation, and all slices were entirely submitted for histologic examination. From each paraffin block, step sections were obtained at 150–200 μm intervals; at each level, 4-μm sections were cut and stained with hematoxylin and eosin for routine evaluation. Cytokeratin immunohistochemistry was not performed routinely and was reserved for cases with equivocal findings on H&E staining when further clarification was deemed necessary. Pathologic reporting, including the total number of sentinel lymph nodes examined, the size of the largest metastatic focus, and the presence of extranodal extension when applicable, was conducted according to established breast cancer reporting standards. Nodal metastasis size categories were defined in accordance with widely accepted contemporary pathology criteria (11). A positive sentinel lymph node was defined as macrometastatic involvement (>2 mm) on permanent section histopathology. Micrometastases (0.2–2 mm) and isolated tumor cells (<0.2 mm) were not classified as nodal metastasis for the purposes of analysis or clinical management. The Research Ethics Committee of Ankara Etlik City Hospital granted ethical approval to our study (AEŞH-BADEK1-2025-566).

We noted down the following variables from the hospital database: age, implant status, tumor size, histopathology, estrogen receptor (ER), progesterone receptor (PR), and human epidermal growth factor receptor 2 (HER2) statuses, tumor grade, presence of lymphovascular invasion, SLNB outcomes, number of SLNs retrieved, number of positive SLNs, presence of extracapsular extension, ALND status, and details of the radiotherapy protocol.

All patients found to have positive sentinel lymph nodes on permanent sections were discussed at a multidisciplinary tumor board. The need for reoperation and completion axillary lymph node dissection was evaluated based on predefined objective criteria, including clinical node-negative status at diagnosis (cN0), hormone receptor–positive/HER2-negative, number and distribution of metastatic sentinel lymph nodes, extent of nodal disease, presence of extracapsular extension, lymphovascular invasion, and eligibility for axillary radiotherapy. Patient-related factors such as age, tumor grade, histological subtype, and comorbidities were considered as secondary modifiers in the multidisciplinary decision-making process. Postmastectomy radiotherapy (PMRT) and axillary radiotherapy were delivered according to multidisciplinary tumor board decisions. In patients with sentinel lymph node metastasis identified on permanent section, completion axillary lymph node dissection was omitted and definitive axillary radiotherapy was administered. Treatment planning was performed using CT-based simulation in the supine position with standard immobilization. Target volumes included the chest wall and regional lymphatic basins. Axillary radiotherapy encompassed levels I–II routinely, with extension to level III and the supraclavicular fossa when indicated based on nodal burden and clinicopathologic risk factors. Internal mammary node irradiation was considered in selected high-risk cases. Radiotherapy was delivered using intensity-modulated radiation therapy (IMRT). A hypofractionated schedule was preferred when feasible (typically 40–42.56 Gy in 15–16 fractions), while institutional organ-at-risk dose constraints for the heart and lungs were respected.

While presenting continuous variables as mean (M) ± standard deviation (SD) and median (minimum–maximum), we summarize categorical variables as number (n) and percentage (%). Normality of distribution was assessed using the Shapiro–Wilk test. We used one-way analysis of variance (ANOVA) and the Kruskal–Wallis test for group comparisons. For categorical variables, we employed Pearson’s chi-square test and Fisher’s exact test when the assumptions of the chi-square test were not met (e.g., when more than 20% of expected cell counts were below 5 or any expected count fell below 1). All analyses were performed using Jamovi version 2.3.28.

## 3 Results

The mean age was 56.7 ± 13.5 years with a median of 58 (range: 26–89). Invasive ductal carcinoma (IDC) was the most prevalent histologic subtype (36, 76%), followed by invasive lobular carcinoma (ILC) in 7 patients (15%) and other histologic types (solid papillary carcinoma in 2 patients and mucinous carcinoma in 2 patients) in 4 patients (9%). The mean tumor size was 29.2 ± 18.8 mm with a median of 25 mm (range: 3–70). Nineteen patients (40.4%) were classified as T1, 21 (44.7%) as T2, and 7 (14.9%) as T3. For histologic grade, 13 patients (27.7%) were classified as Grade 1, 25 (53.2%) as Grade 2, and 9 (19.1%) as Grade 3. All patients consisted of hormone receptor-positive and HER2-negative individuals. All patients underwent axillary staging with SLNB, and none received ALND. Lymphovascular invasion (LVI) was absent in 41 (87.2%). Extracapsular extension was identified in only 2 patients (4.3%). The mean number of SLNs retrieved was 3.77 ± 1.77 (median: 3; range: 1–8).

While 39 (83.0%) were SLN-negative, 8 (17.0%) were SLN-positive. The mean age was 57.4 ± 13.7 years in the SLN-negative group and 53.6 ± 13.2 years in the SLN-positive group (p = 0.484). In both groups, IDC was the most prevalent histopathologic subtype (71.8% vs. 100%, p = 0.229). Mean tumor size did not differ significantly between the groups (28.5 mm vs. 32.9 mm, p = 0.554). In terms of T-stage, the SLN-negative group comprised 38.5% T1, 48.7% T2, and 12.8% T3 tumors. In the SLN-positive group, these rates were found to be 50%, 25%, and 25%, respectively (p = 0.424). Regarding histologic grade, 30.8% of SLN-negative patients were classified as Grade 1, 48.7% as Grade 2, and 20.5% as Grade 3. These rates were found to be 12.5%, 75%, and 12.5%, respectively, in the SLN-positive group (p = 0.390).

Lymphovascular invasion was significantly more prevalent in SLN-positive patients (37.5% vs. 7.7%, p = 0.021). Extracapsular extension was identified in 25% of SLN-positive cases (p = 0.021). The number of SLNs retrieved was comparable between the groups (3.72 vs. 4.00, p = 0.686). In SLN-positive patients, the mean number of metastatic SLNs was 2.0 ± 1.2 (median 1.5; range 1–4). Clinicopathologic characteristics are demonstrated in **Table 1**, and the distribution of sentinel lymph node biopsy (SLNB) status according to the number of removed sentinel lymph nodes and metastatic nodes is presented in **Table 2**. Sentinel lymph node metastasis was identified in 8 patients (17.0%). Among these patients, one patient (2.1%) had 1 of 1 sentinel lymph node positive (1/1), one patient (2.1%) had 2 of 2 positive (2/2), two patients (4.3%) had 1 positive node among three removed (1/3), one patient (2.1%) had 2 positive nodes among four removed (2/4), one patient (2.1%) had 2 positive nodes among five removed (2/5), one patient (2.1%) had 1 positive node among seven removed (1/7), and one patient (2.1%) had 3 positive nodes among eight removed (3/8).

**Table 1:** Patients’ clinicopathological characteristics.

Clinicopathological characteristics	Overall n=47 (%)	Node negative n=39 (%)	Node positive n=8 (%)	P-value
Age Mean Median	56.7±13.5 58 (26-89)	57.4±13.7 58 (26-89)	53.6±13.2 50 (40-77)	0.484
Histopathology IDC ILC Others	36 (76) 7 (15) 4 (9)	28 (71.8) 7 (17.9) 4 (10.3)	8 (100) - -	0.229
Tumor size (mm) Mean Median	29.2±18.8 25 (3-70)	28.5±18.2 25 (3-70)	32.9±22.5 24 (10-70)	0.554
T-stage T1 T2 T3	19 (40) 21 (45) 7 (15)	15 (38.5) 19 (48.7) 5 (12.8)	4 (50) 2 (25) 2 (25)	0.424
Grade Grade 1 Grade 2 Grade 3	13 (27.7) 25 (53.2) 9 (19.1)	12 (30.8) 19 (48.7) 8 (20.5)	1 (12.5) 6 (75) 1 (12.5)	0.390
Lymphovascular invasion No Yes	41 (87.2) 6 (12.8)	36 (92.3) 3 (7.7)	5 (62.5) 3 (37.5)	0.021*
Extracapsular invasion No Yes	45 (95.7) 2 (4.3)	39 (100) -	6 (75) 2 (25)	0.021*
Number of SLNs removed Mean Median	3.77±1.77 3 (1–8)	3.72±1.7 3 (1–8)	4±2.2 3.5 (1–8)	0.686
Number of metastatic SLNs Mean Median	0.34±0.9 0	- -	2±1.2 1.5 (1-4)	<0.001*
Follow-up duration (months) Mean Median	26.8 ± 11.4 27 (1–40)	26.2 ± 11.2 27 (1–40)	29.5 ± 12.0 29 (6–39)	0.580
Chemotherapy No Yes	22 (47) 25 (53)	21 (54) 18 (46)	1 (13) 7 (87)	0.033*
Hormone therapy No Yes	- 47 (100)	39 (100)	8 (100)	–
Radiotherapy No Yes	27 (57) 20 (43)	27 (69) 12 (31)	- 8 (100)	<0.001*

* Statistically significant (p < 0.05).

**Table 2:** SLNB status by number of metastatic and removed SLNs.

SLNB status	n
0/1 0/2 0/3 0/4 0/5 0/6 0/7	4 4 12 9 2 5 3
1/1 2/2 1/3 2/4 2/5 1/7 3/8	1 1 2 1 1 1 1

In patients with two or fewer metastatic sentinel lymph nodes (7 patients, 14.9%), completion axillary lymph node dissection was not recommended following multidisciplinary tumor board discussion. Similarly, in the patient with three metastatic sentinel lymph nodes (1 patient, 2.1%), completion axillary lymph node dissection was not performed, as a total of eight sentinel lymph nodes had been removed and was considered sufficient axillary sampling during multidisciplinary evaluation. None of the sentinel lymph node–positive patients (0%, n = 0) underwent completion axillary lymph node dissection. There were no regional recurrences after a median follow-up of 27 months. The mean follow-up was 26.8 ± 11.4 months, with a median of 27 months (range: 1–40). Follow-up duration did not differ significantly between the SLN-negative and SLN-positive groups (p = 0.58). Adjuvant chemotherapy administration differed significantly between node-negative (n = 39) and node-positive patients (n = 8) (p = 0.033). Chemotherapy was administered to 18 of 39 node-negative patients (46%) and 7 of 8 node-positive patients (87%). All patients in both groups (39/39 and 8/8) received adjuvant hormone therapy; therefore, no statistical comparison was applicable. Radiotherapy was administered to 12 of 39 node-negative patients (31%) and all 8 node-positive patients (100%), demonstrating a statistically significant difference between the groups (p < 0.001).

## 4 Discussion

In this study, we examined whether IFS analysis is essential in patients with cT1–3N0 breast cancer undergoing upfront mastectomy and SLNB. Sentinel lymph node positivity was detected in only 17% of patients, with most SLN-positive patients harboring one or two metastatic sentinel lymph nodes, reflecting a limited nodal disease burden. None of the SLN-positive patients underwent completion axillary lymph node dissection.

This finding can be explained by the fact that all SLN-positive patients were clinically node-negative at diagnosis, exhibited a limited and non-bulky nodal disease burden, and showed no evidence of clinically significant N2–N3 disease. Importantly, all tumors were of hormone receptor–positive/HER2-negative, a group known to have a lower risk of axillary recurrence and high sensitivity to radiotherapy and systemic therapy. Accordingly, axillary management decisions were guided by multidisciplinary tumor board evaluation with planned axillary radiotherapy.

The ACOSOG Z0011 trial in BCS patients and the AMAROS trial in mastectomy patients demonstrated that ALND in those with one or two positive SLNs did not yield significant benefits in terms of survival or local control (4, 12). The findings of these two landmark studies were confirmed by a plethora of previous research (6, 13–18). Thus, the trend toward omitting ALND has become increasingly widespread. In a United States-based study, the rate of ALND decreased from 58% in 2005 to 36% in 2014 (19). Similarly, Hennigs et al. reported that ALND rates in Germany fell from 90% in 2008 to 56% in 2015 (20). This shift away from routine ALND has also led to revisiting the necessity of IFS analysis.

Sargent et al. retrospectively evaluated 623 cT1–3N0 patients who underwent upfront mastectomy and SLNB without IFS analysis. Only 6% of patients required a second surgical intervention with ALND. They noted that the omission of IFS analysis allowed for substantial avoidance of unnecessary axillary surgery (21).

Salman et al. reported that the use of IFS analysis prevented a repeat ALND in only 5% of patients (n = 7) (22). Vongsaisuwon et al. evaluated 265 patients undergoing mastectomy with SLNB; IFS was performed in 202 patients, while 63 were assessed solely via permanent section analysis. The rate of detecting more than three metastatic SLNs was 6/202 (3%) in the IFS group and 1/63 (1.6%) in the permanent section group. Reoperation was required in only one patient in each group (23). In our clinical practice, IFS analysis is not performed for SLNB in clinically SLN-negative patients scheduled for upfront mastectomy. Among eight patients (17%) with positive SLNs upon multidisciplinary review, none underwent ALND; these patients were managed with axillary radiotherapy. Consistent with contemporary practice patterns, axillary management in this patient population has become increasingly conservative. As a result, the routine use of ALND has steadily declined, which in turn diminishes the practical value of intraoperative nodal assessment. In most cases, definitive histopathological evaluation on permanent sections rarely leads to a change in subsequent management, further questioning the necessity of routine intraoperative analysis in this setting. Furthermore, omitting IFS analysis shortens operative time, reduces costs, and minimizes anesthesia exposure (22–25).

It was also demonstrated that performing ALND in patients over 70 years of age with early-stage breast cancer does not contribute to regional or overall survival. In addition, in hormone receptor–positive, HER2-negative patients over 70, SLNB was suggested to be unnecessary (26, 27). In light of these findings, it is conceivable that the role of IFS analysis may be reconsidered for certain patient groups, and SLNB itself could come to be regarded as a historical procedure.

In our study, we extended our current clinical practice in BCS to patients undergoing mastectomy [8]. However, several limitations should be acknowledged, including its single-center, retrospective design and the potential for selection bias. In addition, the sample size was relatively small. Data on long-term oncologic outcomes (e.g., overall survival, local recurrence, and disease-free survival) are not yet available; therefore, future research should evaluate the long-term impact of omitting IFS analysis. The clinical validity of our findings, however, could be enhanced by our basis in real-world clinical practice, individualized patient management guided by multidisciplinary tumor board decisions, and adherence to contemporary guidelines.

## 5 Conclusions

In this retrospective cohort of clinically node-negative, hormone receptor–positive, HER2-negative cT1–3 breast cancer patients undergoing upfront mastectomy, omission of intraoperative frozen section (IFS) analysis was not associated with early regional recurrence during the observed follow-up period. Sentinel lymph node metastasis was detected in 17% of patients. According to contemporary guideline criteria, completion axillary lymph node dissection (ALND) would potentially have been indicated in only one patient (2.1% of the cohort); however, even in that case, ALND was not performed following multidisciplinary tumor board evaluation based on overall clinicopathologic features and the extent of axillary sampling.

Accordingly, routine IFS would have influenced immediate surgical decision-making in only a very small proportion of patients in this selected population. Considering the additional operative time, cost, and resource utilization associated with IFS, its routine use in similar low-risk cohorts may warrant reconsideration. Nevertheless, given the retrospective design, limited sample size, and relatively short follow-up, these findings should be interpreted with caution. Larger prospective studies with longer follow-up are necessary to further clarify the oncologic implications of omitting intraoperative sentinel lymph node evaluation in the upfront mastectomy setting.

## Data Availability

The data analyzed in this study is subject to the following licenses/restrictions: Restrictions apply to the availability of these data due to ethical and legal considerations related to patient privacy. The datasets generated and/or analyzed during the current study are not publicly available but are available from the corresponding author on reasonable request, subject to approval by the institutional ethics committee. Requests to access these datasets should be directed to Bülent Aksel e-mail: akselbulent@gmail.com.
